# [Corrigendum] Palliative radiotherapy in advanced cancer patients treated with immune-checkpoint inhibitors: The PRACTICE study

**DOI:** 10.3892/br.2026.2211

**Published:** 2026-10-06

**Authors:** Melissa Bersanelli, Elisabetta Lattanzi, Nunziata D’Abbiero, Sebastiano Buti, Alessandro Leonetti, Maria Giulia Canè, Salvatore Trapani, Gianluca Gravina, Giampiero Porzio, Katia Cannita, Pietro Di Marino, Antonino Grassadonia, Nicola Tinari, Michele De Tursi, Elisa Giaiacopi, Maria Michiara, Paola Bordi, Fabiana Perrone, Luciana Caravatta, Marianna Trignani, Domenico Genovesi, Clara Natoli, Corrado Ficorella, Marcello Tiseo, Alessio Cortellini

Biomed Rep 12:59–67, 2020; DOI: 10.3892/br.2019.1265

Following the publication of the above paper, the authors and the Journal wish to clarify the ethical and methodological framework of the PRACTICE study. PRACTICE was a secondary retrospective analysis of data collected within the ISyDORe study. ISyDORe was initially approved by the University of L’Aquila IRB, the coordinating centre (approval no. 32865, 24 July 2018). A subsequent Version 3.0 amendment, formally approved by the University of L’Aquila IRB on 11 December 2018 (no. 51/2018.19), incorporated radiotherapy exposure, timing and dose into the study protocol, together with updated informed-consent documentation. Participation of the contributing centres was reviewed according to the applicable local procedures.

Note that this clarification does not affect the study data, analyses, results or conclusions reported in the article.

